# Gastric Cancer in the Setting of Persistently Elevated Human Chorionic Gonadotropin: A Case Report

**DOI:** 10.1155/2011/350318

**Published:** 2011-10-13

**Authors:** LaToya R. Walker, Brian Erler

**Affiliations:** ^1^Department of Obstetrics/Gynecology, Jersey Shore University Medical Center, 1945 Route 33, Neptune, NJ 07753, USA; ^2^Department of Pathology, Jersey Shore University Medical Center, 1945 Route 33, Neptune, NJ 07753, USA

## Abstract

A 35-year-old woman presented to the emergency room for the evaluation of failed surgical and medical management of a suspected ectopic pregnancy. When imaging studies were performed, she had lymphadenopathy and diffuse sclerosis of the osseous framework. Multiple biopsies were performed and revealed poorly differentiated metastatic carcinoma with signet ring features. Esophagogastroduodenoscopy confirmed the findings of a Stage IV gastric adenocarcinoma. Signs and symptoms of gastric carcinoma are vague. However, to our knowledge, an elevation in human chorionic gonadotropin (hCG) is not an associated finding. Persistence of hCG has many causes from abnormal pregnancy to menopause and other forms of cancer.

## 1. Introduction

Elevations in human chorionic gonadotropin (hCG) exist in few conditions outside of pregnancy, such as choriocarcinoma, solid tumors, or perimenopause/menopause [1, 2]. To our knowledge, elevations of hCG have not been associated with gastric adenocarcinoma. The incidence of gastric carcinoma is decreasing worldwide and in the United States. African-Americans, Hispanics, and Native Americans are twice as likely to develop gastric cancer as Caucasians. Etiology remains unknown; however, risks include pernicious anemia, *H. pylori* infection, and/or tobacco usage. Signs/symptoms are nonspecific until advanced disease [3].

We report a case of a 35-year-old female diagnosed with gastric carcinoma in the setting of persistently elevated hCG. She had no evidence of a pregnancy, either normal or abnormal. When imaging studies were performed, she had lymphadenopathy which was biopsied to reveal gastric carcinoma with signet ring cells. Esophagogastroduodenoscopy confirmed the findings, and the patient began chemotherapy for Stage IV gastric carcinoma and no pathological evidence of choriocarcinoma or germ cell component to her tumor. Both urine and serum hCG became negative after chemotherapy.

## 2. Report of a Case

The patient is a 35-year old G4P2012 with a last menstrual period of December 18, 2010 who presented to the emergency room for the evaluation of failed surgical and medical management of a suspected ectopic pregnancy. Approximately 4–6 weeks prior to emergency room visit, patient was treated with methotrexate for asymptomatic ectopic pregnancy with a beta-human chorionic gonadotropin (*β*-hCG) of 54 (0– 5 MIU/mL). After an inappropriate response to medical therapy, a persistent *β*-hCG of 53, she continued to have intermittent bleeding and anemia, for which she received two units of packed red blood cells. After counseling, patient agreed to proceed with laparoscopy and dilation and curettage (D&C). At the time of surgery, there was no evidence of an ectopic pregnancy, and abdominal anatomy appeared grossly normal. The pathology from the D&C showed proliferative endometrium. 

While in the emergency room, the patient's only complaint was bilateral hip pain since her surgery. She denied abdominal pain, vaginal bleeding, nausea, vomiting, or any other symptoms. During evaluation, patient was febrile (temp 101.5), dyspnea, and tachycardiac (120 bpm). Her laboratory values were significant for severe anemia with a hemoglobin/hematocrit of 6.6/29 (hemoglobin: 12–16 gm/dL and hematocrit: 30–48%), alkaline phosphatase of 856 IU/L (38–126), lactate dehydrogenase (LDH) of 959 IU (91–200), and a *β*-hcg of 63 with negative heterophilic antibodies and a positive urine hCG. She underwent a pelvic ultrasound for suspected gestational trophoblastic disease which was normal with an endometrial thickness of 5.8 mm. Further evaluation with a computed tomography (CT) scan of the abdomen and pelvis was significant for retroperitoneal adenopathy along with diffuse sclerosis within the osseous framework. Magnetic resonance imaging (MRI) of spine and bone scan confirmed metastatic malignancy to bony structures of the humorous, ribs, spine, and femur. 

**Figure 1:**
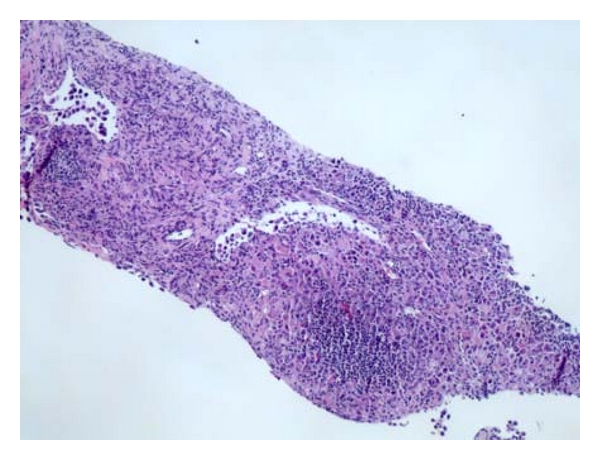
Retroperitoneal lymph node biopsy stained with hematoxylin and eosin (H&E) at 25x magnification showing poorly differentiated carcinoma with signet ring cells.

**Figure 2:**
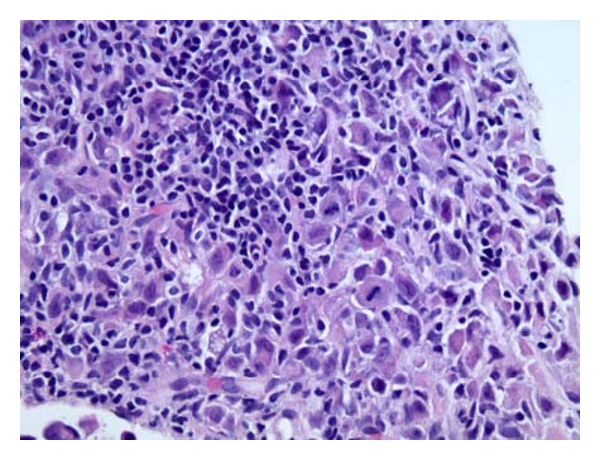
Retroperitoneal lymph node biopsy stained with H&E at 400x magnification showing poorly differentiated carcinoma with signet ring cells.

**Figure 3:**
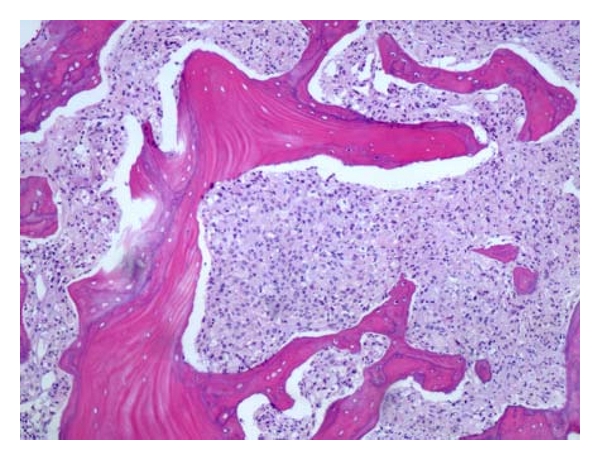
Bone marrow biopsy showing poorly differentiated carcinoma with signet ring cells at 25x magnification and stained with H&E.

**Figure 4:**
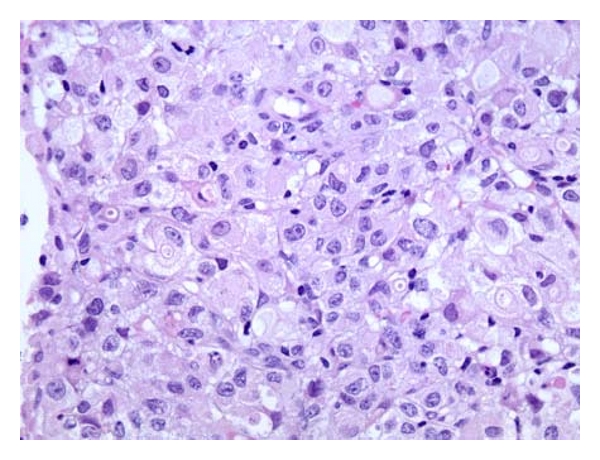
Bone marrow biopsy showing poorly differentiated carcinoma with signet ring cells at 400x magnification and stained with H&E.

**Figure 5:**
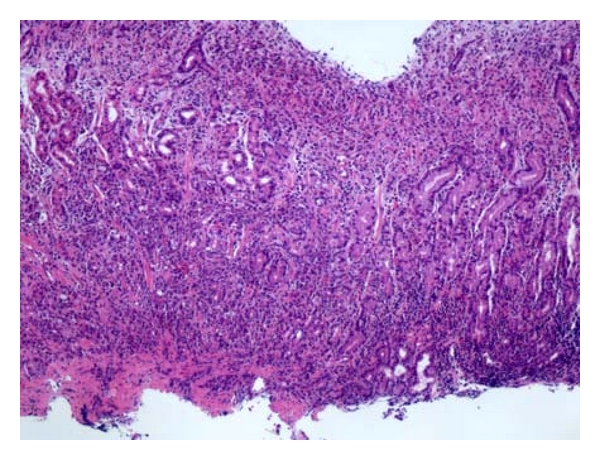
Lesser curvature of the stomach stained with H&E at 25x magnification showing poorly differentiated carcinoma with signet ring cells.

**Figure 6:**
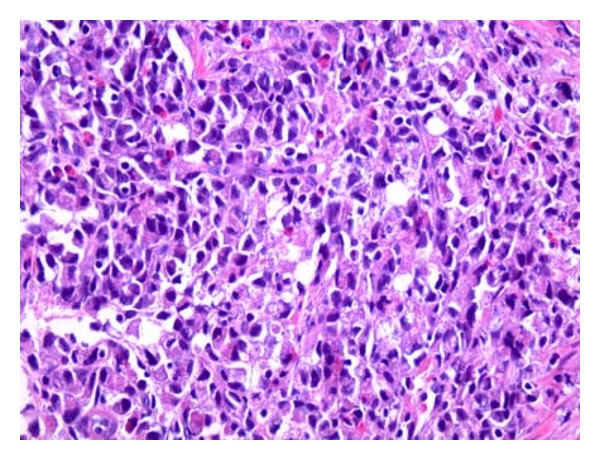
Lesser curvature of the stomach with H&E staining at 400x magnification shows poorly differentiated carcinoma with signet ring cells.

During her hospitalization, she had biopsy of her lymph node, bone marrow and an esophagogastroduodenoscopy (EGD). All of which were positive for gastric carcinoma with signet ring features as shown in Figures 1–6. As shown in Figures 1 and 2 from her retroperitoneal lymph node biopsy, which were stained with hematoxylin and eosin (H&E) at magnifications of 25x and 400x, respectively, demonstrated poorly differentiated metastatic carcinoma with signet ring cells. Biopsy from her bone marrow (Figures 3 and 4) was consistent with the biopsy from her lymph nodes. Stage IV gastric carcinoma (Figures 5 and 6) was confirmed by a biopsy of the lesser curvature of the stomach, which demonstrated poorly differentiated carcinoma with signet ring cells. This patient's physical examination and medical history were unremarkable. She is not a smoker and has no family history of cancer. This patient is currently receiving chemotherapy for Stage IV gastric carcinoma, and her hCG, both urine and serum, has become negative with treatment.

## 3. Discussion

The incidence of gastric cancer has been decreasing worldwide and in the United States. However, in Asia and Eastern Europe, gastric cancer remains the leading cause of cancer death. In the U.S., the average age of occurrence is 70 yrs of age with African-Americans, Hispanics, and Native Americans are twice more likely to develop gastric cancer than Caucasians. Rarely, does this cancer occur before the age of forty. Survival is related to tumor stage, location, and histological features. Twenty percent of gastric cancer is localized. The over-all long-term survival rate is less than 15% [3]. 

The etiology of this disease remains unknown; however, risks for the disease include pernicious anemia, family history, *H. pylori* infection, tobacco usage, and blood group A [4]. The most common neoplasm is adenocarcinoma, which is found in 95% of patients with gastric cancer diagnosis, with the remaining 5% being carcinoid, lymphoma, squamous cell carcinoma, and sarcoma [3]. Tumor grade and histology are important prognostic indicators of this disease. There are several histological classifications, and using the Lauren classification, intestinal which is 53%; diffuse, 33%; unclassified, 14%. Intestinal is usually associated with chronic gastritis, dysplasia, and intestinal metaplasia and is less aggressive. Accounting for approximately 70% of cases and occurs in men and the elderly, with a mean age of 63, the diffuse type usually occurs more commonly in younger patients and carries a poorer prognosis [3–5]. 

Signs and symptoms are usually nonspecific until advanced disease. Patients can complain of dyspepsia, anorexia, and weight loss, early satiety to acute upper gastrointestinal bleeding with hematemesis or melena. Physical examination is rarely helpful because gastric mass is palpate in <20% of cases. Signs of metastatic spread include left supraclavicular lymph node (Virchow node), umbilical nodule (Sister Mary Joseph nodule), ovarian metastases (krukenberg tumor), or rigid rectal shelf (Blumer shelf) [4, 6]. 

Discovered in 1912 by Bernhard Aschner, hCG is the hormone that maintains progesterone production in early pregnancy. It has many roles during pregnancy, such as signaling the uterus to prepare for implantation. Produced by the syncytiotrophoblast cells, it promotes adequate blood supply to the placenta during pregnancy. Elevations in human chorionic gonadotropin (hCG) exist in a few conditions outside of pregnancy, such as gestational trophoblastic disease (GTD) or choriocarcinoma, solid tumors, or perimenopause/menopause. There are three forms of hCG: hyperglycosylated hCG, free *β*-hCG, and pituitary hCG [1]. 

Hyperglycosylated hCG, which is made by cytotrophoblast cells, stimulates implantation and growth of the placenta by promoting invasion and growth of these (cytotrophoblast) cells. Because it stimulates growth and invasion by gestational trophoblastic neoplasms, invasive moles, and choriocarcinomas, it is present in these diseases. Free *β*-hCG is produced by all advanced cancer primaries, such as germ cell tumors, teratomas, leiomyosarcomas, lymphomas, or leukemia. This form of hCG (free) promotes cell invasion and malignancy. Pituitary hCG is naturally produced at extremely low levels during the menstrual cycle. In the perimenopausal and menopausal state, levels of pituitary hCG increase because of the absence of circulating estradiol and progesterone levels [1, 2].

In reproductive aged women with elevated hCG, pregnancy, either normal or abnormal (i.e., missed abortion, ectopic pregnancy, etc.) should be ruled out. Outside of that, malignancy should be ruled out with imaging and blood tests. To our knowledge, gastric adenocarcinoma is not associated with hCG. It is an insidious disease with vague signs and symptoms.
